# Early-Onset Schizophrenia Showed Similar but More Severe Olfactory Identification Impairment Than Adult-Onset Schizophrenia

**DOI:** 10.3389/fpsyt.2020.00626

**Published:** 2020-06-30

**Authors:** Ze-tian Li, Shu-bin Li, Jin-feng Wen, Xiao-yuan Zhang, Thomas Hummel, Lai-quan Zou

**Affiliations:** ^1^ Chemical Senses and Mental Health Lab, Department of Psychology, School of Public Health, Southern Medical University (Guangdong Provincial Key Laboratory of Tropical Disease Research), Guangzhou, China; ^2^ Department of Psychology, Guangdong 999 Brain Hospital, Guangzhou, China; ^3^ Department of Psychiatry, Zhujiang Hospital, Southern Medical University, Guangzhou, China; ^4^ Smell and Taste Clinic, Department of Otorhinolaryngology, Technische Universität Dresden, Dresden, Germany

**Keywords:** early-onset, adult-onset, schizophrenia, odor identification, negative symptoms

## Abstract

**Background:**

“Early-onset schizophrenia” (EOS) is defined as disease with onset before the age of 18 years. This subset of schizophrenia exhibits worse cognitive function and carries a worse prognosis than adult-onset schizophrenia (AOS). Olfactory impairment has been found in patients with schizophrenia-spectrum disorders. However, most research has focused on olfactory impairment in patients with AOS: olfactory function in EOS is not known. The aim of this study was to investigate the olfactory identification ability in EOS, and its relationship with negative symptoms.

**Methods:**

We compared olfactory function between two independent samples: 40 patients with EOS and 40 age- and sex-matched healthy controls (HCs); as well as 40 patients with AOS and 40 age- and sex-matched HCs. The University of Pennsylvania Smell Identification Test was administered.

**Results:**

The EOS group and AOS group exhibited worse olfactory identification ability than HCs; impairment correlated significantly with negative symptoms. Olfactory identification was worse in patients suffering EOS compared with those suffering AOS.

**Conclusion:**

Olfactory identification impairment may be a trait marker of schizophrenia.

## Introduction

Within the sensory system, olfactory neural circuitry is associated most closely with temporo-limbic and frontal-lobe regions. These pathways overlap with brain regions that are typically defective in schizophrenia (1, 2). Several meta-analyses have consistently reported obvious olfactory impairment in patients with schizophrenia, especially in olfactory identification (3, 4). Impairment of olfactory identification has been reported to be related to negative symptomatology in schizophrenia (5–7). In addition, impairment of olfactory identification has been found among first-degree family members of patients with schizophrenia and youths at risk of developing psychosis (4, 7). These aforementioned findings suggest impairment of olfactory function to be a potential trait marker of schizophrenia.

Early-onset schizophrenia (EOS) is a rare, severe, and chronic form of schizophrenia. EOS is defined as disease with an onset before 18 years of age (8, 9). Scholars have reported that patients suffering from EOS exhibit more negative symptoms compared with those with adult-onset schizophrenia (AOS), which is defined as disease with the initial episode presenting after 18 years of age (10, 11). Numerous studies have revealed that patients with EOS show more severe impairment in cognitive function (9, 12), premorbid adjustment (13), and long-term outcome (14, 15) compared with those with AOS. The EOS phenotype is relatively homogenous, and may represent a subgroup with more salient genetic loading less affected by environmental alterations (9, 16, 17). These findings provide a unique opportunity to identify clinically relevant biomarkers in schizophrenia by studying EOS. Most studies regarding olfaction in schizophrenia conducted have focused on patients with AOS.

Only Corcoran and colleagues have examined olfactory function in EOS patients (18). They examined olfactory identification in 26 well-characterized adolescents with early-onset psychosis, including major depression (n = 5), bipolar disorder, mania (n = 3), otherwise unspecified psychosis (n = 5), and schizophrenia or schizoaffective disorder (n = 13). They revealed that deficits in olfactory identification were present in early-onset psychotic disorders (as they are in adults) and were related to cognitive and negative symptoms. However, the sample size was small (only 13 adolescents suffering schizophrenia or schizoaffective disorder). Thus, olfactory identification ability (OIA) in EOS patients is not clear.

Here, we investigated OIA in EOS patients and compared it with that of patients with AOS and healthy controls (HCs). EOS is a more severe phenotype of schizophrenia and shows more impairment in cognitive function compared with AOS, while cognitive function has a strong correlation with olfactory function (19, 20). Hence, we hypothesized that EOS patients and AOS patients would show worse OIA compared with that of HCs, and that impairment would be more severe in EOS patients compared with that in AOS patients. We also examined the relationship between OIA and negative symptoms. We hypothesized that olfactory performance would correlate negatively with negative symptoms in EOS and AOS patients.

## Methods

### Participants

The study protocol was approved by the ethics committees of Southern Medical University and Guangdong 999 Brain Hospital, both of which are in Guangzhou, China. Participants provided written informed consent to be part of this study.

Patients with schizophrenia were recruited from Zhujiang Hospital (Guangzhou, China), Southern Medical University, and Guangdong 999 Brain Hospital. Forty patients (26 males and 14 females; 20.5 ± 2.5 years) who experienced their first episode at age 13–18 years comprised the EOS group. Forty patients (17 males and 23 females; 25.4 ± 5.2 years) who experienced their first episode after the age of 18 years were assigned to the AOS group. The sample size was calculated by G*Power software (21) (*t* tests: difference between two independent means (two groups), two tails, *α* = 0.05, power = 0.8, *d* = −0.97) and the effect size was used according to a previous meta-analysis (5). Patients were diagnosed by trained psychiatrists according to guidelines set forth in *Diagnostic and Statistical Manual of Mental Disorders* (5th edition) (22). The Positive and Negative Syndrome Scale (PANSS) was used to identify clinical symptoms (23). Thirty-four patients of EOS were using antipsychotic medication during the experiment [aripiprazole (n = 7), risperidone (n = 15), olanzapine (n = 10), amisulpride (n = 2), Sertraline (n = 6), paliperidone (n = 10), or quetiapine (n = 5)], and 22 patients of AOS were taking antipsychotics [aripiprazole (n = 1), risperidone (n = 3), olanzapine (n = 8), amisulpride (n = 4), Sertraline (n = 3), paliperidone (n = 5), or quetiapine (n = 3)].

Exclusion criteria were: (i) history of brain injury; (ii) presence of other neuropsychiatric disorders; (iii) history of throat or nose diseases; (iv) severe infection with influenza during the week before study commencement; and (v) history of drug abuse.

Eighty healthy participants recruited from universities and nearby communities were divided into two groups. Participants were matched, respectively, with EOS and AOS patients based on sex and age. Identical exclusion criteria were applied to HC participants. Participants did not have a history of neuropsychiatric disorders. Smoking status was also recorded; only two patients in each patient group had a history of tobacco-smoking, and none had such history in the HC group.

### Olfactory Identification Ability

The University of Pennsylvania Smell Identification Test (UPSIT) is a standardized forced-choice test, and is one of the most widely used smell tests in olfactory research. It is composed of four test booklets, each containing 10 items, for a total of 40 items (24). Each item includes “scratch and sniff” microencapsulated odors attached to a strip at the bottom of the page. The microcapsule was scratched directly by the examiner with a pencil to optimize performance. Subsequently, participants smelled the odor released from the microcapsules and were asked to choose one of four possible responses. If the participant was unsure of the odor emitted, the examiner scratched the patch again. Each correct response was awarded 1 mark. The total score ranged from 0 mark to 40 marks, with a higher score indicating better OIA.

### Self-Reported Hedonic Traits

The Temporal Experience of Pleasure Scale (TEPS) was designed to measure the traits of individuals in anticipatory and consummatory experiences of pleasure (25). We used the Chinese version of the TEPS, which contains 20 self-reported items designed to distinguish the temporal aspects of pleasure. This test was rated using a six-point Likert scale, ranging from 1 (“very false for me”) to 6 (“very true for me”). Higher scores indicated a stronger ability to experience pleasure. The internal consistency of the Chinese version is good, and Cronbach’s *α* of the total scale is 0.83 (26).

### Data Analysis

Comparison of olfactory function (UPSIT) and hedonic traits (TEPS) between patients with EOS, AOS, and their age- and sex-matched HCs was conducted by applying independent *t*-tests using SPSS v22.0 (IBM, Armonk, NY, USA). In addition, we calculated the effect size (Cohen’s *d*) to standardize the group difference of respective olfactory function among two samples (sample A: EOS, HCs; sample B: AOS, HCs) (27). In addition, we carried out a 2 × 2 two-way analysis of covariance (ANCONA) to examine the effects of sex and group on OIA in each sample using education as a covariate. Pearson’s correlation was used to assess the relationship between olfactory function and clinical symptoms (PANSS), age, illness duration, chlorpromazine equivalence (CPZ), and hedonic traits for each group. *P <* 0.05 denoted significance.

## Results

### Participant Characteristics, Hedonic Traits, and Olfactory Test Performance

For the complete sample, significant differences were found between EOS, AOS and sex- and age-matched HC participants. The demographic and clinical data of patients are summarized in **Tables 1** and **2**. Patients in EOS and AOS groups had less educational experience compared with HCs. People in EOS and AOS groups showed significant differences in UPSIT scores compared with HCs (EOS vs. HC: *t*[78] = −5.21, *p* < 0.001; AOS vs. HC: *t*[78] = −2.98, *p* = 0.004). Moreover, the calculated effect sizes of the two samples were medium-to-large [EOS vs. HC: *d_A_* = 1.19, 95% confidence interval (CI) (0.68, 1.63); AOS vs. HC: *d_B_* = 0.66, 95% CI (0.21, 1.11)]. Two-way ANCOVA showed that the main effect of sex was not significant [EOS vs. HC: *F*(1,75) = 3.30, *p* = 0.73; AOS vs. HC: *F*(1,75) = 0.42, *p* = 0.519], whereas the main effect of the group was significant (EOS vs. HC: *F*(1,75) = 9.27, *p* = 0.003; AOS vs. HC: *F*(1,75) = 5.45, *p* = 0.022) after controlling for educational experience. The interaction of sex and group was not significant in sample A or B (*p* > 0.05 for all).

**Table 1:** Demographic and clinical characteristics for participants of Sample A.

	Early-Onset Schizophrenia Patients and Healthy Controls							
	Patients		Healthy Controls					
Variable	(N = 40)		(N = 40)			Analysis		
	Mean	*SD*	Mean	*SD*	*t/χ^2^*	*df*	*p*	
Age (year)	20.48	2.49	20.50	1.91	−0.05	78	0.960	
Education (year)	9.70	2.57	14.10	1.74	−8.96	78	<0.001	
Sex (M:F)	26:14		26:14		0	1	1.000	
Duration of illness (month)	45.68	30.36						
Onset age (years)	16.10	1.15						
Positive and Negative Syndrome Scale								
Positive scale score	18.00	6.50						
Negative scale score	20.68	7.89						
General psychopathology score	40.08	11.93						
CPZ equivalents (mg)	370.60	247.87						
TEPS total	74.35	14.00	87.05	13.85	−4.08	78	<0.001	
TEPS anticipatory	32.70	7.43	37.58	6.92	−3.31	78	0.003	
TEPS consummatory	38.15	8.15	44.85	7.51	−3.82	78	<0.001	
UPSIT	25.13	6.49	31.53	4.27	−5.21	78	<0.001	

SD, Standard Deviation; M, Male; F, Female; CPZ, Chlorpromazine; TEPS, Temporal Experience of Pleasure Scale; UPSIT, The University of Pennsylvania Smell Identification Test.

**Table 2:** Demographic and clinical characteristics for participants of Sample B.

	Adult-Onset Schizophrenia Patients and Healthy Controls						
	Patients		Healthy Controls				
Variable	(N = 40)		(N = 40)			Analysis	
	Mean	*SD*	Mean	*SD*	*t/χ^2^*	*df*	*p*
Age (year)	25.43	5.16	24.13	6.85	0.96	78	0.341
Education (year)	11.37	3.36	15.75	2.23	−6.89	77	<0.001
Sex (M:F)	17:23		13:27		0.85	1	0.356
Duration of illness (month)	36.54	40.15					
Onset age (years)	22.40	4.30					
Positive and Negative Syndrome Scale							
Positive scale score	16.20	6.28					
Negative scale score	16.48	9.32					
General psychopathology score	36.78	13.91					
CPZ equivalents (mg)	254.10	173.40					
TEPS total	73.56	15.10	87.68	12.34	−4.57	78	<0.001
TEPS anticipatory	32.38	7.69	38.08	6.30	−3.63	78	0.001
TEPS consummatory	37.38	9.03	45.50	7.11	−4.47	78	<0.001
UPSIT	29.35	4.20	32.13	4.14	−2.98	78	0.004

SD, Standard Deviation; M, Male; F, Female; CPZ, Chlorpromazine; TEPS, Temporal Experience of Pleasure Scale; UPSIT, The University of Pennsylvania Smell Identification Test.

Patients with EOS showed a significantly lower score in negative symptoms (*t* [78] = 2.18, *p* = 0.033) compared with those with AOS. However, there were no significant differences in positive symptoms or general psychopathology (*p* > 0.05 for all). CPZ and illness duration did not differ between EOS and AOS groups (*p* > 0.05 for all).

As shown in **Tables 1** and **2**, samples A and B differed significantly in terms of TEPS total score (EOS vs. HC: *t*[78] = −4.08, *p* < 0.001, *d_A_* = 0.91, 95% CI (0.45, 1.37); AOS vs. HC: *t*[78] = −4.57, *p* < 0.001, *d_B_* = 1.02, 95% CI (0.55, 1.49), respectively), TEPS anticipatory subscale score (EOS vs. HC: *t*[78] = −3.31, p = 0.003, *d_A_* = 0.74, 95% CI (0.28, 1.19); AOS vs. HC: *t*[78] = −3.63, *p* = 0.001, *d_B_* = 0.68, 95% CI (0.36, 1.27), respectively), and TEPS consummatory subscale score (EOS vs. HC: *t*[78] = −3.82, *p* < 0.001, *d_A_* = 0.85, 95% CI (0.39, 1.31); AOS vs. HC: *t*[78] = −4.47, *p* < 0.001, *d_B_* = 1.00, 95% CI (0.53, 1.46).

### Correlational Analysis

**Figure 1:**
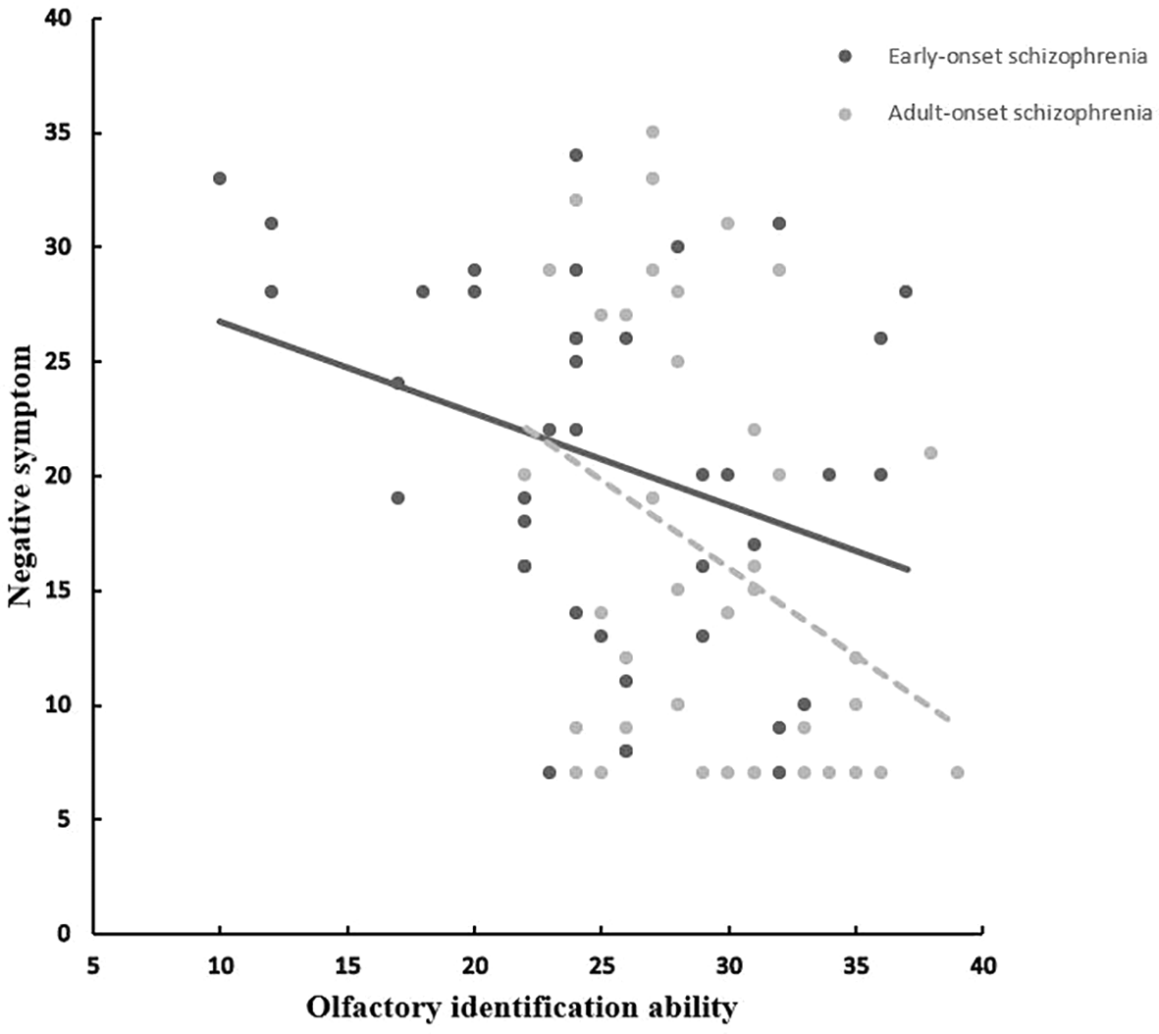
Correlation between olfactory identification ability (UPSIT) and negative symptom in EOS and AOS groups. UPSIT, University of Pennsylvania Smell Identification Test; EOS, Early-onset schizophrenia; AOS, Adult-onset schizophrenia.

The OIA of EOS patients correlated significantly with negative symptoms (*r* = −0.331, *p* = 0.037) (**Figure 1**). All clinical-subscale scores of patients with AOS correlated significantly with olfactory function (positive symptoms: *r* = −0.409, *p* = 0.009; negative symptoms: *r* = −0.345, *p* = 0.029; general psychopathology: *r* = −0.372, *p* = 0.018). Moreover, the olfactory function of HCs in sample B correlated significantly with the TEPS anticipation subscale score (*r* = 0.379, *p* = 0.016). There were no significant relationships between olfactory function and total or subscale TEPS scores in sample-A HCs and patients with EOS or AOS (*p* > 0.05). A significant correlation between age and olfactory function in the EOS group (*r* = −0.334, *p* = 0.035) was documented. In both patient groups, OIA was not related to age of onset, illness duration, or CPZ (*p* > 0.05).

## Discussion

Here, we aimed to evaluate impairment of olfactory identification in patients with EOS and AOS. Such impairment has been reported in schizophrenia but the age of onset has not been addressed. This was the first study to investigate OIA in adults with EOS.

Our initial hypotheses were confirmed because patients suffering EOS or AOS exhibited significantly worse olfactory identification compared with sex- and age-matched HCs. These findings are in accordance with those of studies that consistently reported obvious olfactory impairment in patients with schizophrenia (3, 4). Furthermore, comparison of the effect size with regard to olfactory-identification impairment revealed differences between EOS (*d* = 1.19) and AOS (*d* = 0.66), suggesting impairment of greater severity in the former group of patients. In addition, results revealed that male patients did not differ from female patients with respect to OIA in the EOS group or AOS group, a finding that is consistent with that in other studies (28–30). Notably, Mossaheb et al. (31) found that female patients performed better than male patients in the olfactory-identification test, but the effect was only borderline significant (31).

Notably, there was no difference between patients with EOS and AOS in terms of illness duration or CPZ. Hence, the worse OIA observed in EOS compared with AOS may be attributed (at least in part) to brain maturity. Numerous studies have reported gray-matter volume in the frontal and temporal lobes of patients suffering EOS to be reduced (32–34). Matsumoto (32) reported a positive correlation between the volume of the bilateral superior temporal gyrus and age at disease onset. Although the findings of a longitudinal study revealed that the pattern of abnormal cortical thickness in EOS is similar to that observed in AOS, lower volume has been observed consistently in prefrontal and temporal cortices (35). These brain regions have a crucial role in olfactory function.

We found that only negative symptoms were more severe in EOS compared with AOS. Our findings are consistent with those of other studies, indicating that patients suffering EOS exhibit more negative symptoms than those with AOS (10, 11). In addition, our findings revealed that EOS and AOS patients experienced less pleasure than HCs. These findings are also in accordance with recent data from Zou and colleagues for patients with first-episode schizophrenia and individuals with schizotypy, who were of a similar age to the people evaluated in the present study. They revealed that the patient group suffered a significant loss of pleasure compared that in HCs (7). However, a significant difference in experiencing pleasure among patients suffering EOS and AOS was not observed.

Correlation analyses revealed a significant negative correlation between negative symptoms and OIA in EOS and AOS patients. In contrast with studies investigating individuals with schizotypy and patients with first-episode schizophrenia (7, 36), a significant association between olfactory function and self-reported experience of pleasure in chronically ill patients suffering EOS and AOS was not found. Several scholars have reported OIA to be associated with negative symptoms in adult patients with schizophrenia (5–7). Our findings are consistent with those reported by Corcoran et al. (18), who detailed a relationship between negative symptoms and OIA in early-onset psychosis. Corcoran et al. (18) stated that impairment of olfactory identification is a trait-like marker of vulnerability to schizophrenia and the manifestation of negative symptomology. A possible explanation for this phenomenon is that negative symptoms that occur during childhood and adolescence may affect acquisition of the language skills necessary to identify odors correctly (18).

Interestingly, our study did not reveal a correlation between the age of onset and reduced OIA in patients with EOS or AOS. This observation may be explained by the narrow range of the age of onset in both patient groups (EOS: 16.1 ± 1.5 years; AOS: 22.4 ± 4.3 years). In addition, reduced OIA did not correlate with illness duration or CPZ in either patient group. These findings suggest that the observed OIA reduction reflected olfactory function in schizophrenia itself.

Our study had several limitations. First, we recruited only adults with EOS. Future research should also investigate olfactory function in children and adolescents with EOS. Second, Seckinger suggested that the intelligence quotient (IQ) was related to OIA in schizophrenia (37). However, we did not test IQ due to time constraints, so whether olfactory impairment was because of IQ impairment in EOS was not investigated. Future studies must address this issue. Even so, OIA showed impairment in EOS and AOS after controlling for the educational level in our study. Third, we only assessed OIA in patients with EOS versus those with AOS. Future research should investigate olfactory function in a more differentiated way; that is, adding olfactory threshold and odor-discrimination tests using the Sniffin’ Sticks test (38). In addition, we only recruited EOS and AOS patients according to their first episode information in our study; future study should provide information about other episodes. Finally, this was a cross-sectional study; further longitudinal research is warranted to validate our findings.

In summary, we found the EOS group and AOS group exhibited worse OIA than HCs, and the impairment was correlated significantly with negative symptoms. Furthermore, OIA was worse in patients suffering EOS compared with those suffering AOS. With previous research revealed the olfactory identification impairment in first-episode schizophrenia and individuals with schizotypy (7), this continuity suggests olfactory identification impairment may be a trait marker of schizophrenia and emphasizes the significance of early diagnosis and intervention. Also, special olfactory treatment (e.g., olfactory training) may decrease the negative symptoms in schizophrenia, and future study need to address this issue.

## Conclusions

EOS is linked to a more severe deficit in olfactory identification compared with AOS and is associated with the manifestation of negative symptoms. Our findings support olfactory identification impairment may be a trait marker of schizophrenia and also provide a new insight into the early intervention for schizophrenia.

## Data Availability Statement

The datasets generated for this study are available on request to the corresponding author.

## Ethics Statement

The studies involving human participants were reviewed and approved by the ethics committees of Southern Medical University and the Guangdong 999 Brain Hospital. The patients/participants provided their written informed consent to participate in this study.

## Author Contributions****

Z-TL helped design and run the study, analyzed, and interpreted the data, and wrote the manuscript. S-BL recruited the participants, helped run the study, analyzed, and interpreted the data. J-FW recruited the individuals with schizophrenia, provided demographic and symptom information, and helped with manuscript preparation. X-YZ helped with manuscript preparation and provided comments on the final version. TH helped with manuscript preparation and provided comments on the final version. L-QZ helped design and run the study, analyzed and interpreted the data, and wrote the manuscript. All authors contributed to the article and approved the submitted version.

## Funding

This work was supported by the National Natural Science Foundation of China (grant number: 31700963), the Natural Science Foundation of Guangdong Province, China (grant number: 2017A030310069; 2019A1515012135), and the Medical Science and Technology Foundation of Guangdong Province, China (grant number: 201906546). These funding agents had no further role in any aspect of the study or the writing of this paper.

## Conflict of Interest

The authors declare that the research was conducted in the absence of any commercial or financial relationships that could be construed as a potential conflict of interest.
